# Towards Collaborative Autonomous Operations in Power Infrastructure: A Robotic Fine Manipulation Framework

**DOI:** 10.3390/s26185877

**Published:** 2026-09-17

**Authors:** Guangda Xu, You Dong

**Affiliations:** Department of Civil and Environmental Engineering, The Hong Kong Polytechnic University, Hong Kong SAR, China; guangda.xu@connect.polyu.hk

**Keywords:** robot operating system (ROS), collaborative autonomy, small-scale component detection, fine manipulation, infrastructure operation

## Abstract

Gas-insulated substations (GISs) have been widely adopted in modern power systems due to their compact design and high reliability. However, the potential generation of toxic byproducts poses significant risks to manual operations such as gas pressure adjustments, highlighting the necessity for robotic deployments. This paper proposes a Robot Operating System (ROS)-based robotic framework with human–robot collaborative autonomy for GIS operations. The framework employs a 6-degree-of-freedom (6-DOF) robotic arm, integrated with a motion control system for trajectory execution, a visual perception system enhanced by the coordinate attention (CA) mechanism for small-scale detection and localization, and a communication system for data exchange. The framework improves the accuracy of component perception and enables fine manipulation in complex environments, reducing reliance on manual intervention while facilitating a safe collaborative autonomous workflow through dynamic adjustment of autonomy level and control authority. Experimental results on the representative gas pressure adjustment task demonstrate an autonomous operational success rate exceeding 90% under the proposed configuration in dynamic scenarios. By enhancing safety and precision, this study advances robotic solutions for hazardous operations and lays a foundation for broader infrastructure applications.

## 1. Introduction

Gas-insulated substations (GISs), characterized by compact design, low current losses, and high reliability, have been widely deployed in modern power transmission networks. Typically, a GIS employs sulfur hexafluoride (SF6) as an insulating medium. However, under partial discharge or high temperatures, SF6 can be decomposed into toxic byproducts including sulfur tetrafluoride (SF4) and sulfur dioxide (SO2) [1], posing risks of dizziness, unconsciousness, or fatality upon inhalation, especially in poorly ventilated and confined areas such as control rooms [2]. Such environments highlight the need for enhanced safety and efficiency in GIS operations.

Traditionally, GIS operational tasks, such as gas pressure adjustment, required human operators to work in hazardous conditions. While advanced sensors and imaging technologies have improved the detection of faults, these systems have primarily focused on data collection rather than direct intervention due to the complexity of precise manipulation and control [3,4]. Recent advances in robotics, particularly in autonomous systems and fine manipulation, have provided promising solutions for GIS management and maintenance [5,6].

Robotic technologies have been extensively employed in infrastructure inspections across various industries, including energy, transportation, and civil engineering. Unmanned ground vehicles (UGVs) and unmanned aerial vehicles (UAVs) have demonstrated flexibility in inspecting transmission lines, bridges, and tunnels. Equipped with visual sensors and LiDAR, UAVs have been utilized to detect anomalies in hard-to-reach areas [7,8,9]. For ground-level tasks, UGVs have been proven effective for thermal and visual inspections, equipment fault detection, and gas leakage localization [10,11]. Despite these advancements in inspection, robotic systems for operational tasks, such as gas pressure adjustment in GISs, remain unexplored due to the requirements of fine manipulation.

This study proposes a Robot Operating System (ROS)-based robotic framework for GIS operations, addressing challenges in small-scale object detection, precise manipulation, and seamless human–robot communication. Given its high frequency in routine maintenance and the precision required for both perception and manipulation, the gas pressure adjustment task is adopted as a representative and safety-critical case for demonstration and evaluation, executing the complete perception–communication–manipulation pipeline. Specifically, in the framework, a motion control system is implemented for kinematics computation, motion planning, and collision detection. To enhance the detection and localization of small components such as gas valves, a coordinate attention (CA)-enhanced efficient YOLOv8 network for improved small-scale object detection is integrated. In addition, a communication system is developed to provide interaction channels among different robotic systems and support the collaborative autonomy paradigm, in which the robot executes routine steps autonomously while dynamically transferring control authority to the human operator when necessary, providing an efficient and reliable balance between autonomy and supervision. The proposed framework enables the robotic arm to detect and manipulate gas valves with a high degree of autonomy in GIS environments, demonstrating potential for broader applications in infrastructure maintenance and offering a safe and efficient alternative to manual operations.

The key contributions of this study are as follows.

1. We develop a ROS-based robotic framework for collaborative autonomous GIS operations. The framework integrates a visual perception system optimized for the task scenario, a motion control system for precise manipulation, and a communication system for efficient human–robot collaboration (HRC) and data exchange into a unified platform.
2. We implement a collaborative autonomy paradigm, which supports dynamic HRC by enabling autonomous execution with on-demand remote intervention and control authority transition, improving framework reliability and reducing operator workload.
3. We propose an enhanced YOLO-based detection network integrating a CA module, significantly improving the detection of small-scale components in complex operational environments.
4. Through extensive experiments, we evaluate the performance and adaptability of the proposed robotic framework, demonstrating its potential to improve efficiency and safety in relevant tasks and facilitate the advancement of industrial robotics.

This paper is organized as follows. Section 2 reviews related research on robotics for infrastructure applications, as well as relevant vision models and ROS-based frameworks. Section 3 details the methodology, including the environment setup and the mechanisms of visual perception, motion control, and communication. Section 4 presents the experimental results and relevant discussion, followed by conclusions and future work in Section 5.

## 2. Related Work

### 2.1. Robotic Systems for Infrastructure Applications

Infrastructure such as substations, which serve as critical components of power networks, requires periodic inspection and management to ensure functional reliability. Robotic platforms, including UAVs and UGVs, have been widely deployed across various scenarios to enhance inspection efficiency and accuracy while minimizing human exposure to hazards. For example, Deng et al. [4] and Langåker et al. [7] deployed UAVs for aerial inspection of transmission lines and substations. With their compact size and aerial perspectives, UAVs were also utilized in hard-to-reach areas such as tunnels and towers [8,12]. Some aerial robots were equipped with monocular or binocular cameras, infrared sensors, and LiDAR to detect faults, including insulator contamination and structural damage [9,13,14].

In comparison, UGVs are suited for ground-level inspection tasks due to their stability and high payload capacity. Equipped with vision sensors and LiDAR, UGVs have been deployed for inspections in power plants, bridges, and tunnels [3,15,16]. Lu et al. [10] reviewed UGVs for ground-based inspections of substations, which navigate along predefined paths to capture thermal and visual data of components. Kumar et al. [11] developed a ground mobile robot for gas source localization to prevent leakage incidents. Lim et al. [17] utilized a UGV-based robot for concrete crack detection, while Nguyen et al. [18] equipped a UGV with cameras, ground-penetrating radar (GPR), and electrical resistivity (ER) sensors for overall assessment of structural health conditions.

Recent studies have also advanced the performance analysis and control of robotic systems. For instance, Shen et al. [19] proposed a Bayesian inference-assisted reliability analysis framework for robotic motion systems, and Liu et al. [20] developed a fixed-time neural control scheme for robots under input-saturation and prescribed-performance constraints. Additionally, Sinico et al. [21] compared PID and model-based control strategies for trajectory-tracking performance on open PLC-based robot controllers, highlighting the impact of control schemes on the capabilities of robotic automation. These works demonstrate the importance of reliability assessment and control strategies in robotic deployments.

Despite these advancements, most robotic systems in infrastructure settings have primarily focused on data collection and inspection rather than physical operations, which are often characterized by complexity and variability, requiring fine and precise manipulation and control [22]. Recent developments in robotic arms and control algorithms have enabled new possibilities for the deployment of robots in operational tasks [5]. In this work, we develop a robotic platform for executing manipulation tasks in substations, rather than being limited to inspections. This study has the potential to advance robotic operational solutions to broader applications, thereby enhancing the efficiency and reliability of various civil infrastructures.

### 2.2. Vision Models for Small-Scale Component Detection

Precise and efficient detection of target components is critical for robotic operations. Contemporary detectors are typically categorized into two-stage and one-stage approaches. Two-stage detectors, such as Faster R-CNN [23] and Cascade R-CNN [24], can achieve high accuracy but may suffer from slower inference [25,26], while one-stage detectors, such as YOLO [27,28], have been widely adopted for real-time processing in engineering practice. For example, the YOLO algorithm has been applied for concrete corrosion detection to assist in civil infrastructure inspections [29]. Some studies have also applied YOLO for insulator defect and rusted component identification [30,31]. Additionally, transformer-based models, such as Real-Time Detection Transformer (RT-DETR) [32], capture long-range dependencies and contextual information, improving object localization through self-attention [33], and have been utilized for bridge crack and pavement inspection [34,35].

However, gas valves, switches, and connectors in substations often appear as small-scale objects within the working environment, occupying few pixels in captured images and hindering effective feature extraction [36]. Given the requirement for real-time inference, complex models cannot be a feasible solution for this issue. While the existing detection models, including the YOLO series and DETRs, have demonstrated satisfactory performance in general object detection, their effectiveness on such small components remains suboptimal [37,38].

In recent years, attention mechanisms have attracted interest for enhancing feature representation in detection networks, and several designs have been proposed with different exploitation approaches to spatial and channel information. Traditional channel attention, such as Squeeze-and-Excitation (SE) [39], reweights channel-wise feature responses according to global pooling statistics but does not explicitly model spatial positioning. This idea was extended by the Convolutional Block Attention Module (CBAM) [40], which sequentially generates a channel attention map and a spatial attention map to refine features along both dimensions, and by Efficient Channel Attention (ECA) [41], which avoids reducing the channel dimension through local interaction across channels to lower complexity while preserving channel relationships. At the kernel level, Selective Kernel (SK) networks [42] adaptively fuse multi-branch features with different receptive fields via softmax attention, capturing scale variability for objects of differing sizes, although the fusion weights are shared across all spatial positions. More recently, CA [43] was proposed as a lightweight mechanism that embeds positional information by splitting global pooling into two separate pooling steps, encoding spatial relationships along the horizontal and vertical directions. In contrast to SE and ECA, which only model dependencies among channels and do not explicitly encode spatial cues, and to CBAM, which generates a spatial map through a local operation with limited interaction over long distances, CA captures dependencies over long ranges along each spatial direction while preserving explicit coordinate information along the orthogonal direction. In this study, we enhance YOLOv8 by integrating the CA mechanism as an adaptive augmentation to improve small-scale component perception while maintaining real-time inference capabilities, enabling reliable detection and localization of GIS components, thereby facilitating accurate robotic operations.

### 2.3. ROS-Based Frameworks

ROS has emerged as a standard middleware for robotic applications and has been widely implemented across various infrastructure scenarios [44]. ROS-based frameworks play a crucial role in the development and validation of robotic systems. For instance, they have been utilized for deep learning-based visual perception and navigation for autonomous ground vehicles [45], UAV navigation in cluttered environments integrating vision-based localization and obstacle avoidance algorithms [46], robotic grasping with reinforcement learning policies [6], and multi-robot collaboration and exploration [47]. However, there remains a gap in the development of a comprehensive framework that fully integrates robotic operations for GISs.

This study develops a comprehensive ROS-based framework for autonomous operations in GISs, integrating a CA-enhanced perception system for small-scale component detection, a motion control system for precise and efficient command execution, and a communication system for data interaction and feedback. By unifying these components, the framework provides an extensible and flexible platform for developing and executing autonomous operational strategies.

As an overview, the representative related studies are summarized in Table 1.

## 3. Methodology

**Figure 1:**
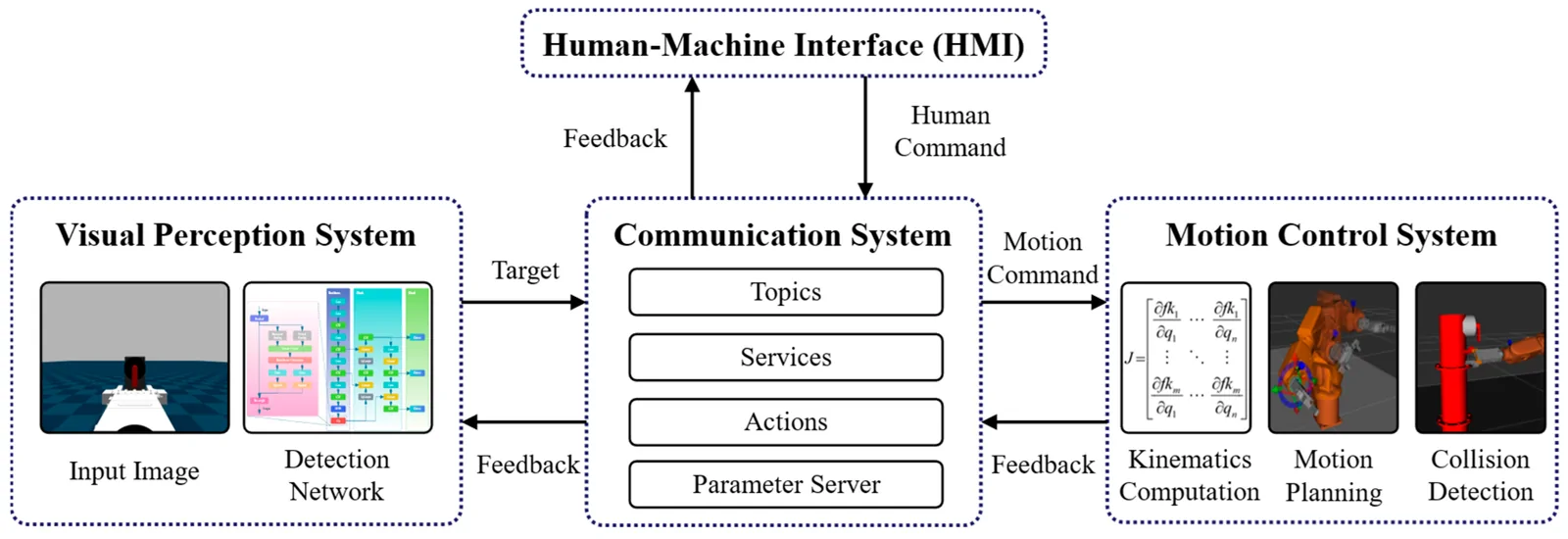
Overall architecture of the proposed robotic framework.

The proposed robotic framework consists of four primary components, as illustrated in Figure 1. Specifically, first, we design and implement a virtual operational environment, which provides an intuitive platform for data collection, framework execution, and performance evaluation. Second, we establish a motion control system primarily based on inverse kinematics, motion planning, and collision detection, enabling precise handling and execution of operational commands. Third, we develop an enhanced visual perception system, in which a CA module is integrated into the backbone of the YOLO network to improve the performance of object detection. Lastly, we implement a communication system for the framework, facilitating seamless collaborative autonomy and data exchange. This section provides a comprehensive explanation of the incorporated environment and systems in the framework.

### 3.1. Environment Setup

This section introduces the design and implementation of the virtual operational environment, which provides a realistic operational setting for data collection and serves as a controlled testbed for evaluating the performance of the proposed framework.

In this study, Gazebo is utilized for assembling a customized operational scenario following the Unified Robot Description Format (URDF). The complex components, including the robotic arm and gas valve, are modeled using 3D mesh files [48], encoded in standard triangle language (STL) and digital asset exchange (DAE) formats, and defined in the form of link-joint structures. Xacro (XML Macros) is employed to simplify URDF configuration through a modular approach, enabling seamless integration of new components for further development.

**Figure 2:**
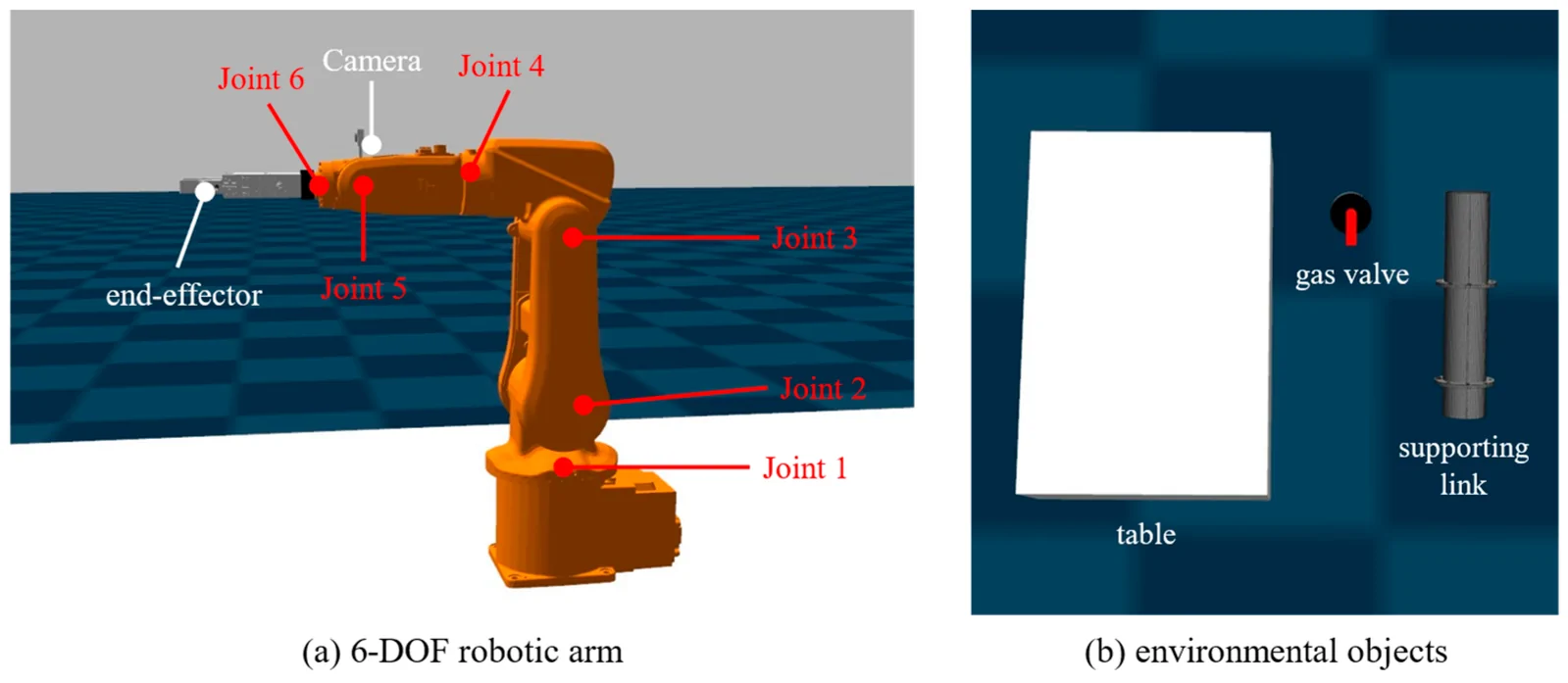
Elements in the virtual operational environment.

As shown in Figure 2, the simulation environment mainly consists of a 6-degree-of-freedom (6-DOF) robotic arm and GIS-related environmental objects. The robotic arm configuration allows for flexible positioning and orientation of the end-effector, while the environmental components include a gas valve assembly, a supporting link, and a table that together constitute part of the GIS setting. To enable realistic actuation of the gas valve, the valve body is modeled as a rigid link connected to the fixed supporting structure via a rotational joint. The physical resistance and mechanical constraints of the valve are modeled through the dynamics and limit properties in the Open Dynamics Engine (ODE). For smooth validation of the perception–communication–manipulation pipeline, the properties are configured to ensure the required manipulation torque remains within the capability of the end-effector setup. During actual deployment, selection of hardware configurations should be performed based on the torque requirements of the target valve specifications. The manipulation is performed through position control, where the achievement of the target angular position rather than the applied torque determines task completion.

Without loss of generality, the ABB IRB 120 robot is selected for experimental demonstration due to its agility, compactness, and lightweight properties, which make it well-suited for the planned fine manipulation tasks. The robotic arm consists of six rotational joints, and the end-effector is equipped with a SCHUNK EGP 64 gripper for securing and adjusting operations on gas valves, as shown in Figure 2a. The framework is compatible with various arms, and the experiments so far have confirmed smooth operation with IRB 120 and UR3e robots.

To ensure the perception capability, an RGB-D camera is mounted on the fifth link of the robotic arm, providing a first-person view (FPV) of the execution area. The camera is attached via a fixed joint, minimizing collision and relative displacement risks and ensuring consistent coordinate calibration. The camera specifications mainly include a frame resolution of 640 × 480, a frame rate of 30 FPS, and an operating range of 0.05 m to 3 m. Alternative sensors with different specifications can be integrated through the modular configurations according to specific task requirements. The transformation between the camera and the end-effector coordinate frame is calibrated for accurate spatial positioning and manipulation, supporting monitoring and intervention during execution.

Overall, the virtual operational environment provides an adaptable platform for validating and refining autonomous operational strategies in GIS settings. The modular nature of the environment allows for further expansions, such as additional sensors or robotic arms, for broader engineering applications.

### 3.2. Motion Control

The overall performance of the framework depends heavily on the ability to precisely interact with and operate in complex environments. This section presents the methodologies of the motion control system in the proposed framework, focusing on the mechanisms for inverse kinematics (IK) computation, motion planning, and collision detection.

#### 3.2.1. IK Computation

To simplify user interaction and facilitate collaborative autonomous operations, all goal requests sent by human users and action clients are specified with respect to the end-effector frame. Consequently, before executing any motion, the framework first computes appropriate joint angles for the arm according to the desired end-effector position and orientation, which are fundamental for precise manipulation.

For this purpose, we employ the Kinematics and Dynamics Library (KDL) as the primary solver, utilizing the Newton–Raphson method with joint limit constraints to iteratively solve the following system of nonlinear equations:

(1) $$f\;=\;\left[\begin{array}{c} fk_{1}\left(q_{1},\cdots {,q}_{n}\right)\;-\;p_{goal1}\\ {fk}_{2}\left(q_{1},\cdots {,q}_{n}\right)\;-\;p_{goal2}\\ \begin{array}{c} \vdots \\ {fk}_{m}\left(q_{1},\cdots ,q_{n}\right)\;-\;p_{goalm} \end{array} \end{array}\right]\;=\;\left[\begin{array}{c} 0\\ \begin{array}{c} 0\\ \vdots \end{array}\\ 0 \end{array}\right]$$

where m represents the dimension of the operational space, n is the number of actuated joints of the robotic arm, and q denotes the joint angles to be determined; ${fk}_{m}$ denotes the forward kinematics function, which maps joint angles to the end-effector pose, while $p_{goalm}$ represents the target point of the end-effector in the corresponding dimension of the workspace. For the IRB 120 model, n = 6, which corresponds to the six rotational joints of the robotic arm, and m = 6, representing the three translational DOFs (x, y, z) and three rotational DOFs (roll, pitch, yaw) of the task space. Therefore, the robotic arm is non-redundant as n = m. Replacement with a higher-DOF robotic arm will result in kinematic redundancy (n > m), increasing flexibility while introducing additional computational demands.

To solve the IK equations in Equation (1), the Jacobian matrix is computed as in Equation (2), and joint angles are updated using the iterative formula in Equation (3).

(2) $$f^{\prime }=J=\left[\begin{array}{ccc} \frac{\partial {fk}_{1}}{\partial q_{1}} & \cdots & \frac{\partial {fk}_{1}}{\partial q_{n}}\\ \vdots & \ddots & \vdots \\ \frac{\partial {fk}_{m}}{\partial q_{1}} & \cdots & \frac{\partial {fk}_{m}}{\partial q_{n}} \end{array}\right]$$

(3) $$\left[\begin{array}{c} q_{1}^{k+1}\\ q_{2}^{k+1}\\ \begin{array}{c} \vdots \\ q_{n}^{k+1} \end{array} \end{array}\right]=\left[\begin{array}{c} q_{1}^{k}\\ q_{2}^{k}\\ \begin{array}{c} \vdots \\ q_{n}^{k} \end{array} \end{array}\right]-J^{-1}e_{k}$$

where e represents the error between the current and target end-effector poses. The Moore–Penrose pseudo-inverse $J^{+}$ is substituted when the Jacobian is not invertible. The iteration is repeated until the solution q converges within a position error tolerance of $10^{-6}$ m, or is terminated after 0.05 s or 100 Newton–Raphson iterations, with an error message returned for operator judgment to maintain computational efficiency.

#### 3.2.2. Motion Planning and Collision Detection

To facilitate effective motion planning, we implement a versatile planning interface supporting various algorithms. In this work, the rapidly-exploring random trees-connect (RRT-Connect) algorithm is adopted for trajectory generation, which iteratively extends two trees from the initial and goal positions through random exploration to form a valid motion path. The planning process terminates upon reaching 5000 iterations or a time limit of 5 s. In this study, the step size for joint space search is configured as 0.1 rad. To accelerate convergence while avoiding local minima, we introduce a 10% probability of expanding directly toward the target instead of sampling a random pose. These parameters were selected to ensure planning efficiency while maintaining the quality of solutions and can be reconfigured for adaptation to other task scenarios.

Collision avoidance is a critical aspect of robotic motion to ensure the robotic arm does not interfere with itself or environmental objects. In the proposed framework, the Flexible Collision Library (FCL) is employed to handle collision detection, and the Bounding Volume Hierarchy (BVH) is utilized for efficient computation, where axis-aligned bounding boxes (AABBs) and oriented bounding boxes (OBBs) are generated for each mesh link.

In this study, both self-collision between different parts of the robotic arm and full collision between the robot and external obstacles or objects are considered. Such comprehensive collision awareness is essential for safe operation during intricate movements. To optimize efficiency, an allowed collision matrix is adopted to exempt predefined link pairs from collision checks if they are physically unlikely to collide. In this study, the matrix is generated by sampling points and further supplemented by the authors. For instance, adjacent links connected via rotational joints and distant links are excluded, reducing computational demands and improving planning efficiency.

Overall, by implementing the kinematics computation, motion planning, and collision detection mechanisms, the motion control system ensures precise and smooth execution of robotic operational tasks.

### 3.3. Visual Perception

The visual perception system in the proposed robotic framework serves to detect the gas valves from visual input and to determine the optimal approaching point for robotic manipulation. This system plays a crucial role in ensuring precise operation by enabling real-time perception and localization of target components in complex environments. Given the challenges associated with small-scale object detection, we enhance a YOLO-based detector by integrating a CA module to improve accuracy. This section details the integration of the CA mechanism and the coordinate transformations from the image frame to the robot’s operational space.

#### 3.3.1. Integration of Coordinate Attention

The proposed framework employs a YOLO-based vision network for gas valve detection in the workspace. As reviewed in Section 2.2, while YOLO is recognized for its accuracy and efficiency in general object detection, it encounters limitations when applied to small-scale components such as gas valves in GISs, which occupy few pixels in captured images, leading to limited feature extraction when incorporated into the framework.

To address this limitation, we propose to employ YOLO as the foundation of the visual perception network and integrate the CA mechanism [43], which embeds positional information into channel attention, improving the representation of relevant objects while maintaining computational efficiency. Through this integration, the sensitivity of the perception system to fine-grained features of components can be enhanced, thereby improving the accuracy of localization and identification.

**Figure 3:**
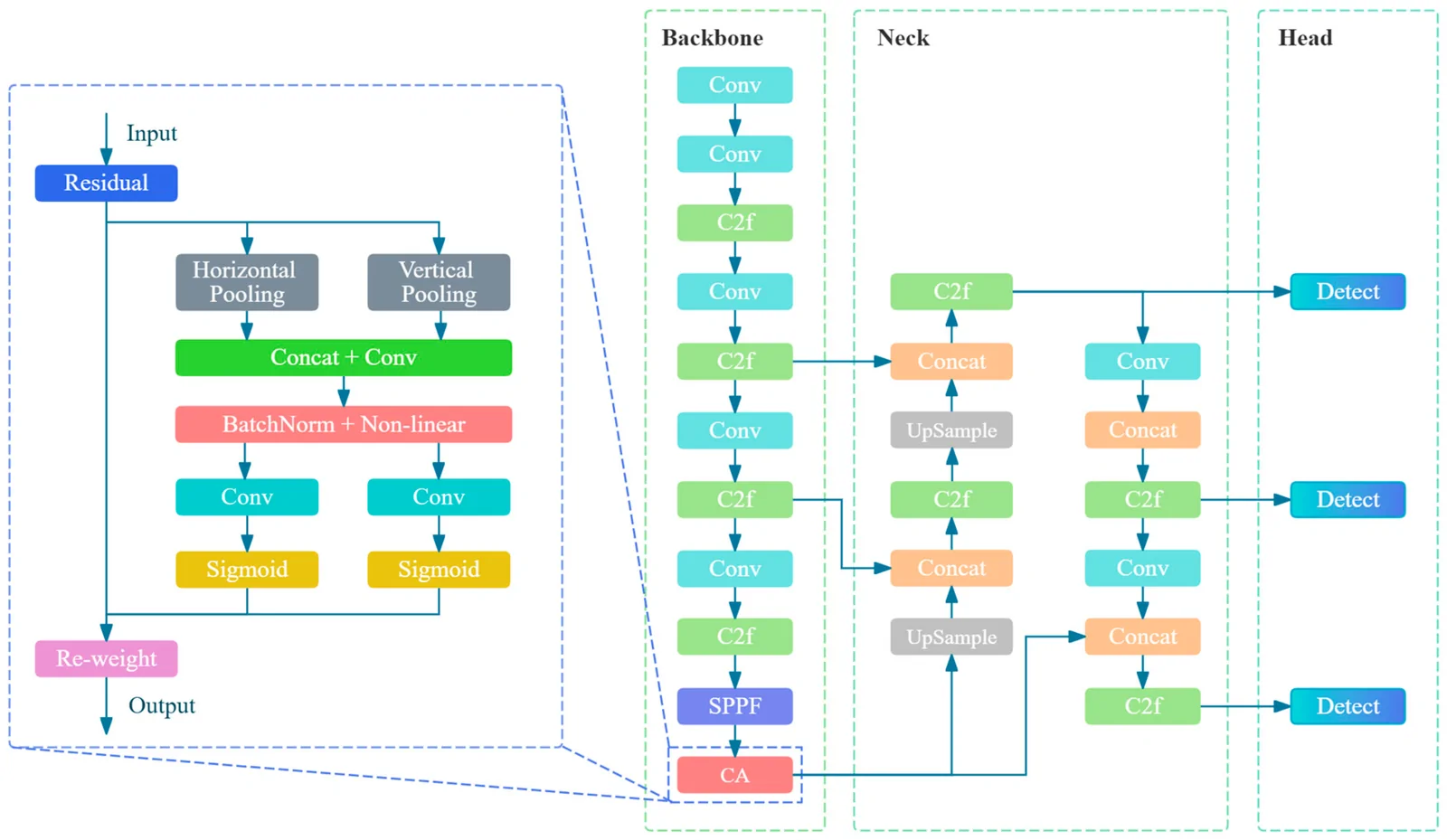
Architecture of the proposed YOLOv8-CA network.

In this study, we insert a CA module into YOLOv8, positioning it as the last layer in the backbone, as shown in Figure 3. This placement is crucial as premature applications might act on relatively raw features, while late applications might lead to the loss of fine-grained spatial details necessary for localizing small targets. Specifically, the CA module operates by separately aggregating visual input features along the vertical and horizontal axes to generate two attention maps, where each element encodes the positional relevance and reflects the presence of the interested object in the corresponding row and column. Subsequently, the attention maps are applied to the intermediate feature tensor through multiplicative weighting, outputting a transformed tensor with enhanced feature representations while preserving spatial integrity. At this stage, the features passed to the subsequent neck structure are already enhanced with attention-guided information. Through this integration, we intend to direct the vision model to attend to the critical spatial regions and features relevant to small-scale objects before proceeding to feature fusion, thereby enabling better localization of gas valves in cluttered backgrounds. Consequently, the enhanced network, referred to as YOLOv8-CA, is expected to outperform the original YOLOv8 and achieve higher performance within the proposed framework.

#### 3.3.2. Coordinate Extraction and Transformation

When the YOLOv8-CA network detects a gas valve, the visual perception system transforms the detected bounding box coordinates from image space into robotic operational space to enable precise grasping through coordinate extraction, camera calibration, and multi-step coordinate transformations.

**Figure 4:**
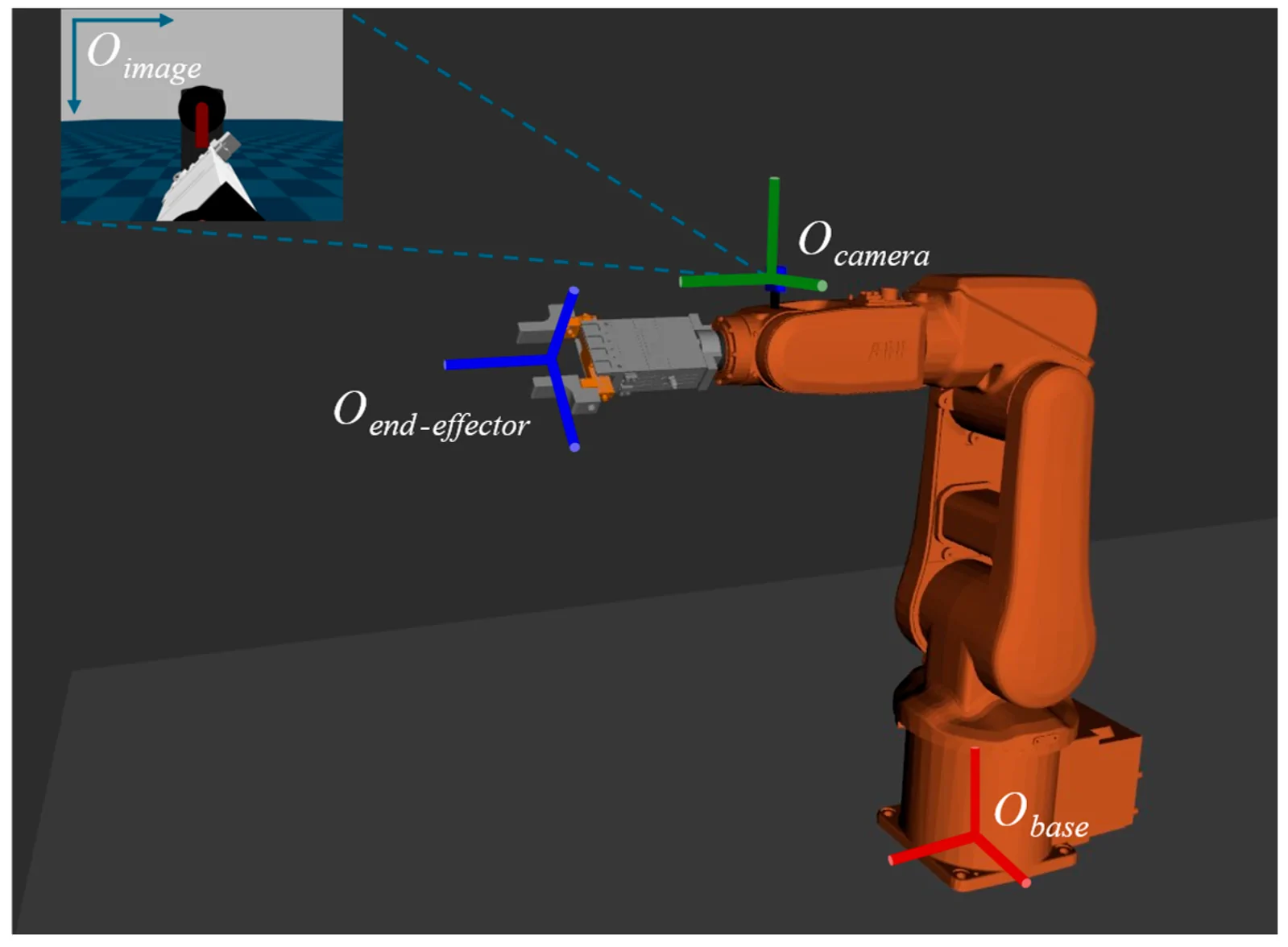
Transformation from image coordinates to robot end-effector coordinates.

Specifically, the vision network first predicts and outputs a bounding box around the detected gas valve, from which the center grasping point $P_{i}$ is extracted in image coordinates. Then, using the camera intrinsic parameters, a transformation matrix $T_{I}^{C}$ is established, which converts pixel coordinates into the camera coordinate system, where the resulting point is denoted as $P_{c}$. Lastly, based on robot model parameters, two additional transformation matrices, $T_{C}^{B}$ and $T_{E}^{B}$, are derived, which describe the translation and rotation of the camera and end-effector frames with respect to the base link of the robot, respectively. These transformations enable the conversion to $P_{e}$ in the end-effector frame. The complete transformation is defined by Equation (4) and illustrated in Figure 4.

(4) $$P_{e}=\left[\begin{array}{c} x_{e}\\ y_{e}\\ z_{e} \end{array}\right]=T_{B}^{E}\cdot T_{C}^{B}\cdot T_{I}^{C}\cdot P_{i}={\left(T_{E}^{B}\right)}^{-1}\cdot T_{C}^{B}\cdot T_{I}^{C}\cdot \left[\begin{array}{c} x_{i}\\ y_{i}\\ z_{i} \end{array}\right]$$

The aforementioned transformations ensure accurate mapping of detected gas valve coordinates into the robot’s operational space, enabling precise manipulation of the robotic arm. Notably, in the proposed framework, the visual perception system is designed as an independent ROS package, allowing convenient integration and replacement of detection models. Future updates and alternative vision models can be seamlessly integrated without modifying the core framework. This modular feature enhances the scalability and versatility of the framework.

By integrating a CA-enhanced vision model and a precise coordinate transformation pipeline, the visual system provides improved perception capabilities, ensuring accurate and reliable detection and localization.

### 3.4. Communication Paradigm

Robust and efficient communication is essential for coordinating robotic subsystems and ensuring seamless data exchange, command execution, and real-time feedback. The framework employs a modular communication architecture, where nodes continuously exchange sensor outputs, control commands, and feedback signals to ensure smooth and coordinated operations. Serving as the backbone for collaborative autonomy, this system enables dynamic adjustment of execution autonomy levels, allowing for seamless human intervention when necessary. The proposed framework incorporates multiple ROS-based communication mechanisms, including topics, services, parameters, and actions, each serving specific roles in handling different types of data interactions.

#### 3.4.1. Topic-Based Communication

**Figure 5:**
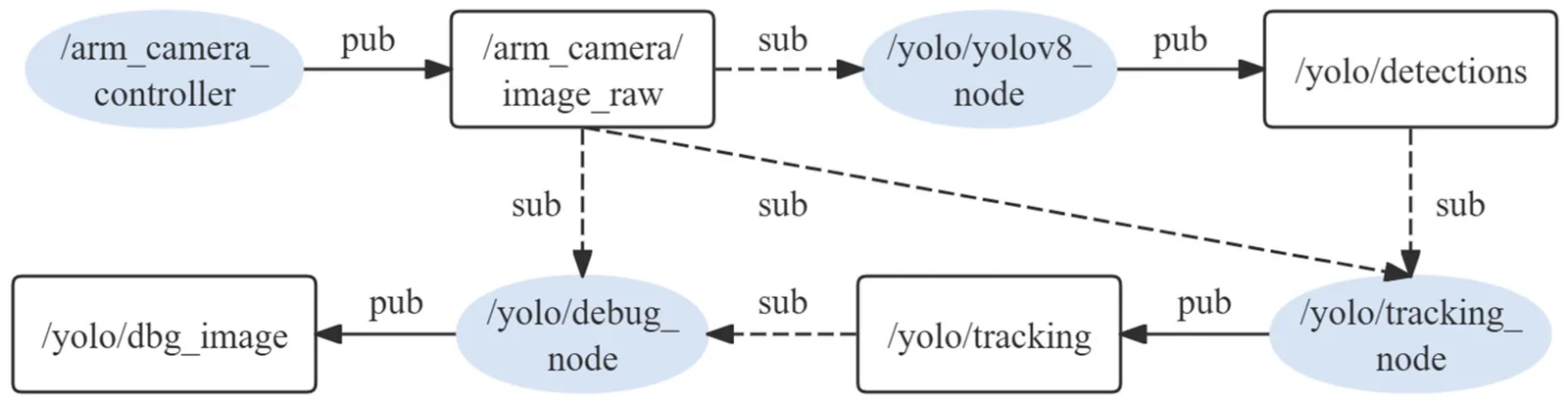
Topic-based communication for visual perception.

**Figure 6:**
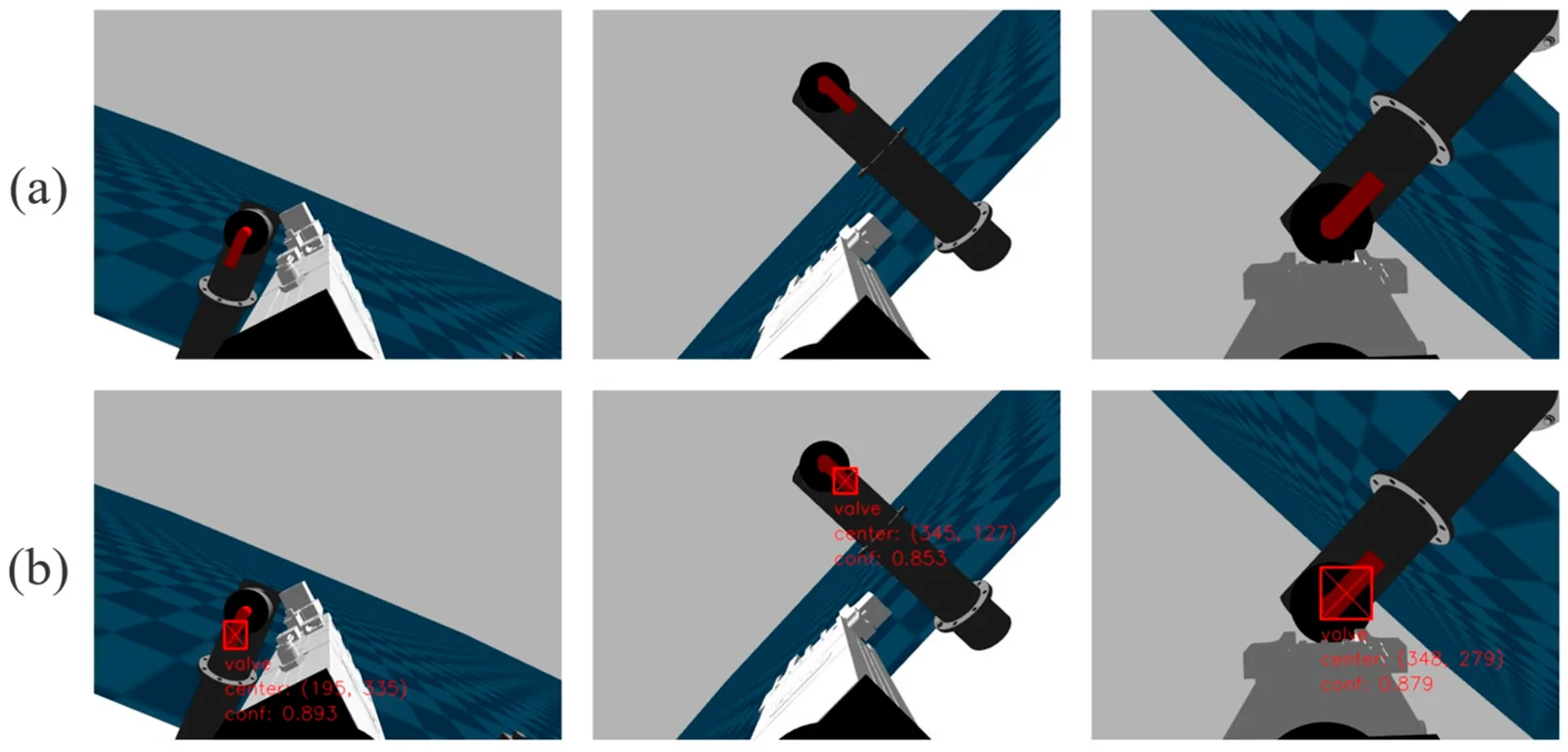
Visualization examples of topics: (**a**) /arm_camera/image_raw and (**b**) /yolo/dbg_image.

The topic-based communication follows a publisher–subscriber paradigm for continuous data streams. The primary application in the framework is the transmission of visual data from the camera to the YOLOv8-CA detection node, as shown in Figure 5, where nodes are represented as ellipses and topics as rectangles. The ‘pub’ with solid lines and ‘sub’ with dashed lines denote the publishing and subscribing streams, respectively. Specifically, raw images are published from the camera node /arm_camera_controller to the topic /arm_camera/image_raw, which is subscribed to by /yolo/yolov8_node for detection. The detection results, including the bounding boxes and confidence scores, are published to /yolo/detections, with /yolo/tracking maintaining continuous tracking in dynamic scenes. Lastly, the /yolo/debug_node publishes annotated images to /yolo/dbg_image for visualization, with examples provided in Figure 6.

The topic-based mechanism allows multiple nodes to publish or subscribe to a single topic as needed, facilitating flexible integration of additional processing modules. Multi-subscriber topics provide a unified environment representation for human operators and robotic modules, improving transparency and interpretability in collaborative autonomy.

#### 3.4.2. Service-Based Communication and Parameter Configuration

**Figure 7:**
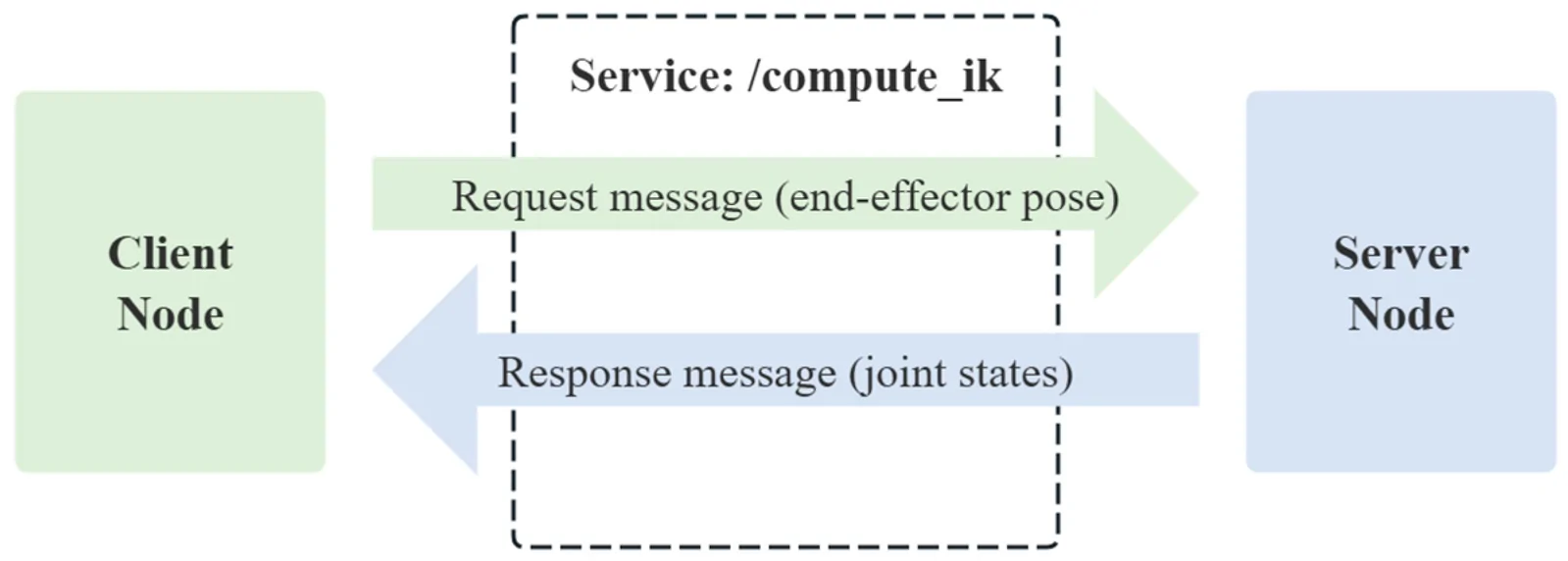
Service-based communication for IK computation.

Different from topic-based communication that follows one-way data flows, service-based communication operates on a request–response paradigm, which is suitable for on-demand queries and commands, particularly those with logical processing requirements. In the proposed framework, the main application is the IK solver, as illustrated in Figure 7. To invoke this service, a client node from the motion planning module sends a request containing the desired end-effector pose, motion constraints, and timestamp. The server then processes the request and returns the joint values required to achieve the target end-effector pose. In the proposed framework, this service is typically invoked during motion planning processes. Compared to unidirectional and continuously streamed topics, service-based communication is bidirectional, ensuring each request receives a corresponding response, making it ideal for complicated queries.

Similar to the idea of distributed architectures, each node in the framework maintains independent parameters that can be stored, retrieved, and modified dynamically during runtime, without restarting nodes. For instance, the camera node stores the frame rate at which the camera generates image data, and the node for coordinate system transformations is configured with the transformation settings between the corresponding parent and child frames. The parameters, stored as key–value pairs, allow users to fine-tune system behavior without stopping or recompiling the framework. The parameter inherits the lifecycle from the node it belongs to and can be dynamically modified through service invocations, enhancing the flexibility of system configuration and improving the adaptability to different operational conditions.

#### 3.4.3. Action-Based Communication

**Figure 8:**
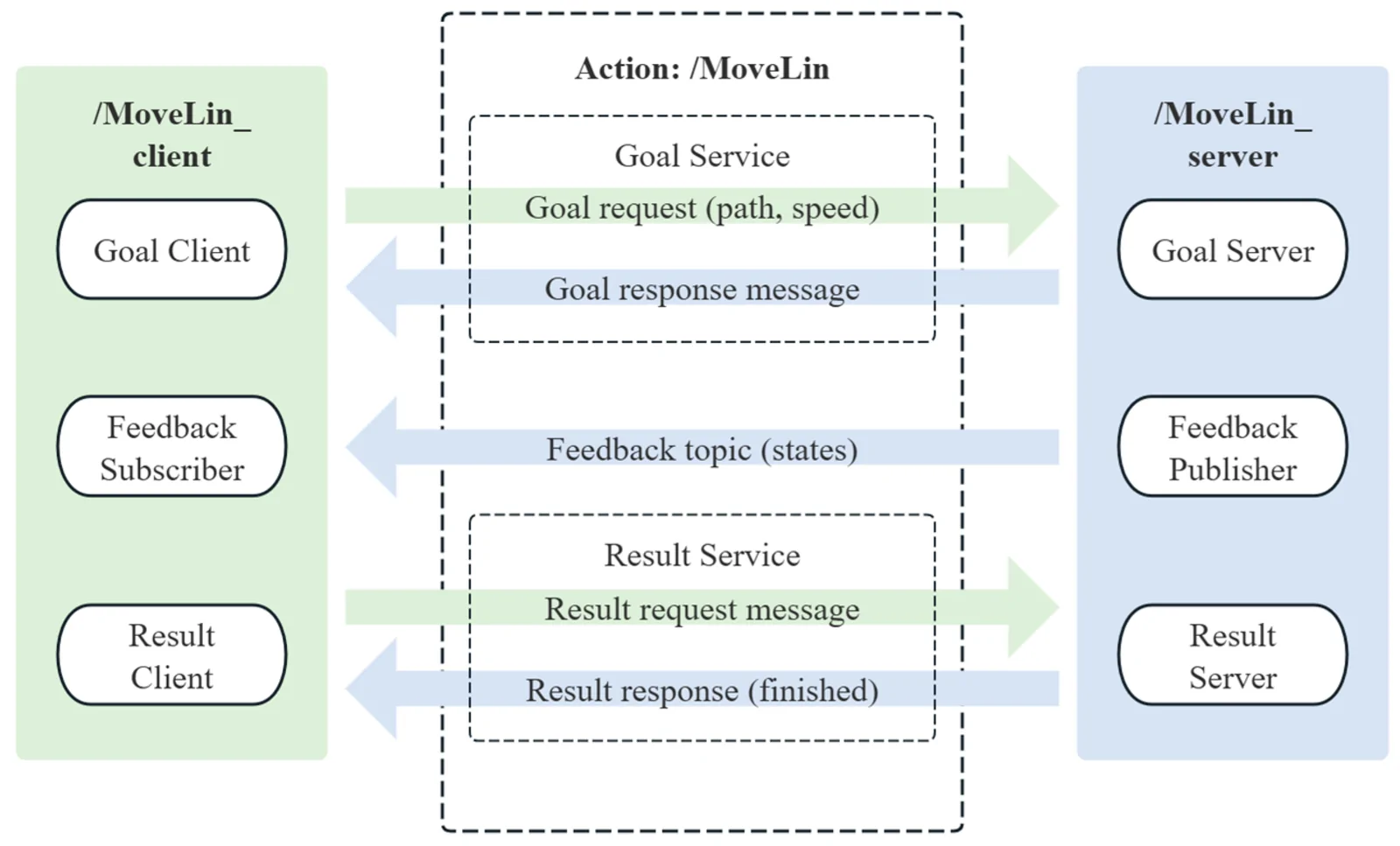
Communication mechanism of the /MoveLin action.

Since robotic motion typically involves long-duration tasks, action-based communication provides real-time feedback and the ability to intervene during execution, which is critical for safe collaborative autonomy. The mechanism primarily operates through the exchange of goal, feedback, and result messages. Specifically, an action client first sends a goal request, specifying the target or path, movement speed, and other necessary parameters. The server then provides continuous updates on the status, allowing users to monitor the execution progress or send additional requests. Upon completion, the server returns the final execution result for confirmation. To facilitate the issuance of commands through human–machine interfaces (HMIs) such as the command line, a series of predefined action commands is implemented, as shown in Table 2, which serve as operator-facing verbs in the paradigm of collaborative autonomy. As an illustration, the communication flow of the /MoveLin action is visualized in Figure 8. The framework also supports defining action sequences of arbitrary length and executing them as an integrated unit. Through action-based communication, the framework ensures real-time monitoring and smooth execution of robotic operations.

#### 3.4.4. Collaborative Autonomy

Existing robotic systems for infrastructure operations can be broadly categorized into teleoperation-based systems, where human operators command all actions in real time, resulting in high cognitive load and latency issues, and pre-programmed fully autonomous systems, where robots operate independently but lack situational awareness and adaptive intervention capabilities needed in complex and unpredictable environments [49]. The collaborative autonomy paradigm implemented in this framework operates as a process that dynamically allocates control authority between the robot and the human operator based on situational uncertainty. This section details the trigger conditions and implementation logic for autonomy level transition and control authority transfer.

The framework continuously monitors three uncertainty indicators during autonomous execution, including the detection confidence score from the visual perception system, planning validity from the motion controller, and manipulation deviation from the feedback signals. The perception-related trigger for authority transfer occurs when the detection confidence falls below the predefined threshold, indicating insufficient reliability to proceed autonomously. Other triggers include planning failures, such as when the RRT-Connect planner fails to find a feasible trajectory within the iteration or time limits, and manipulation deviations, such as when unexpected collisions are detected or when the end-effector pose deviates significantly from the predetermined path. When any of these conditions are met, the robot immediately suspends its autonomous sequence and raises a human intervention request via the HMI.

During task execution, the human operator can monitor the perception and motion control data remotely, including detection results, real-time sensor readings, and planned trajectories. Upon receiving an authority transfer request, the HMI presents the task state, the intervention reason, and relevant sensor data. The operator can then take over teleoperation through the predefined action commands listed in Table 2. Once the operator confirms that the situation is resolved or the task is completed, execution authority is returned to the robot. In addition to on-demand transfer, the framework also supports proactive autonomy level transition, where the operator can issue commands directly at any point during execution. This strategy ensures that the robot handles routine and low-uncertainty steps autonomously while the human operator intervenes only when necessary, thus balancing safety and reliability.

Overall, the proposed framework integrates multiple communication mechanisms to facilitate efficient data exchange, real-time feedback, and collaborative autonomy, achieving seamless HRC and intra-framework coordination, thereby ensuring safety, efficiency, and reliability.

## 4. Experiments and Discussion

### 4.1. Experimental Setup

To evaluate the proposed framework, we conducted a series of experiments for a systematic assessment of the gas pressure adjustment task in GIS operational settings. To ensure the consistency and reliability of evaluation, each trial followed a specified sequence as follows.

(1) Initialization and perception. The robotic arm was reset to a predefined configuration, as shown in Figure 2a, with the first two links perpendicular to the base and the others parallel. The visual perception system then captured RGB-D images for gas valve detection. The confidence score and bounding box determined the execution feasibility and the optimal approaching point, which was transformed from pixel coordinates to the operational space for spatial localization.
(2) Motion command computation and verification. The framework computed a joint motion command to position the end-effector at the target location and orientation. Before execution, the command was displayed in the HMI for human-in-the-loop verification to reduce potential errors and enhance transparency. For rigorous evaluation, trials failing user verification were recorded as failures.
(3) Command execution. The verified motion command was transmitted to the robotic arm for execution. After reaching the target pose, the gripper secured the valve component and rotated it to a specified angle for gas pressure adjustment.

The proposed framework was evaluated under two separate settings. In the first setting, the robot was placed at a fixed and randomly selected position without prior knowledge, evaluating the consistency of outputs and robustness against random noise through iterative execution. In the second setting, the relative position between the arm and the gas valve was randomly sampled from a uniform distribution within the range specified in Equation (5) for each trial, evaluating the adaptability under realistic operational conditions.

(5) $$\left\{\begin{array}{c} x_{T}-x_{A}\in \left[0.35,\;0.55\right]\;m\\ y_{T}-y_{A}\in \left[-0.15,\;0.15\right]\;m\\ z_{T}-z_{A}\in \left[-0.20,\;0.15\right]\;m \end{array}\right.$$

where $x_{T,A}$, $y_{T,A}$, and $z_{T,A}$ stand for the x, y, and z coordinates of the target and the end-effector of the robotic arm, respectively.

**Figure 9:**
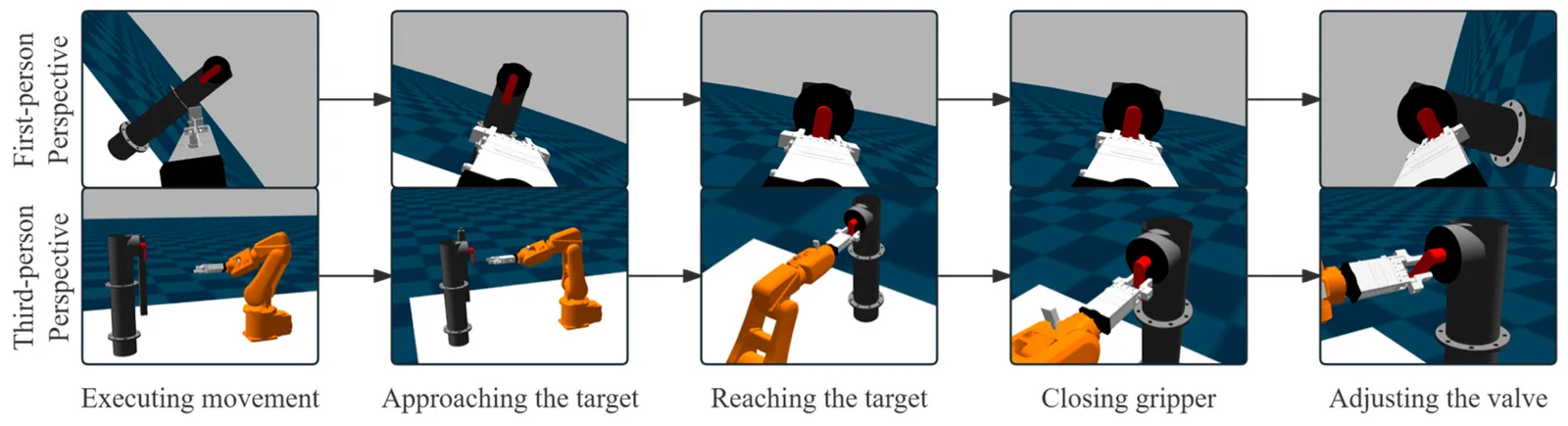
First-person and third-person views of the execution process during an experiment.

Each trial was supervised and rigorously assessed throughout the evaluation. Since the gripper fingers move synchronously, slight horizontal deviations could result in eccentricity and insufficient holding force, while vertical deviations could introduce additional torque requirements, increasing the difficulty of manipulation. As experimentally tested, to ensure smooth operations, the horizontal and vertical coordinate deviations of the grasping point should be limited to 2 mm and 15 mm, respectively, based on the component dimensions and gripper constraints. Figure 9 presents the execution process during an experiment from first-person and third-person perspectives. The experiments were designed to comprehensively evaluate the framework’s capability in detecting target components, as well as inferring and executing manipulations in the operational environment.

It should be noted that the experiments are designed to evaluate the integrated pipeline from perception to manipulation and the overall performance of the proposed framework. While Gazebo provides a controlled testbed for this validation, several factors should be considered when migrating to physical hardware deployment. Calibration errors such as residual hand–eye calibration bias, while controllable in simulation, may become notable in physical systems due to mounting offsets and mechanical vibrations. Additionally, communication and control latencies are also controlled in simulation but may introduce additional delays in practical deployments. The complex and dynamic lighting fluctuations in physical settings are also difficult to fully replicate during vision model training, posing challenges to perception. To address these gaps, domain randomization, synthetic-to-real fine-tuning, and physical deployment have been planned as future directions for this work. The collaborative autonomy paradigm also enables mitigation of such deployment discrepancies by transferring control to human operators when necessary.

### 4.2. Vision Model Evaluation

In this section, comparative experiments were conducted to evaluate the proposed YOLOv8-CA detection model against several baseline models, including YOLOv5-CA, YOLOv8, YOLOv5, and RT-DETR. Ablation studies were also performed to assess the proposed architectural enhancement. This evaluation aimed to analyze the impact of CA integration on small-scale component detection, thereby assessing the overall performance of the visual perception system.

For the purpose of model training, a dataset consisting of 664 images extracted from the virtual operational environment was established. The images had a resolution of 640 × 480 and were manually annotated using LabelImg. The dataset was divided into training (70%), validation (20%), and testing (10%) subsets. Several data augmentation techniques, including random rotation, color jittering, and Gaussian noise injection, were applied to enhance the robustness and generalization capability of the models.

Based on the analysis presented in Section 3.3.1, a CA module was embedded at the final stage of the YOLOv8 backbone. To validate this architectural decision, ablation studies were conducted by varying the insertion position of the CA module, where the adopted configuration was benchmarked against multiple variants, as shown in Table 3.

It is worth mentioning that the CA module operates on the feature tensor without changing the number of channels. Here, P3, P4, and P5 denote the three multi-scale feature levels of YOLOv8, corresponding to the outputs passed to the neck. In the neck, the upsampling path denotes the pathway from P5 to P3 via upsampling and concatenation, and the downsampling path refers to the reverse pathway via downsampling and concatenation. It can be seen from the table that the proposed insertion at the final backbone stage achieves the highest mAP50-95 among all configurations, whereas the F1-score remains within a narrow band of 0.88–0.92 across variants. In this context, since mAP50-95 computes the mean of average precision (AP) across a range of intersection over union (IoU) thresholds, it is more sensitive to localization precision and thus serves as the more discriminative metric; F1-score, in contrast, primarily reflects basic detection performance, capturing whether objects are correctly detected rather than how precisely they are localized. Additionally, our analysis indicates that embedding CA at shallow stages, such as the early feature levels P3 and P4 with smaller receptive fields, may not provide sufficient semantic information for the attention mechanism to leverage. Meanwhile, deploying CA across multiple stages does not bring significant mAP50-95 advantages over the proposed single placement and reduces inference speed to some extent, suggesting that redundant attention computation is performed. Consequently, the proposed single insertion at the final backbone stage provides the most favorable balance between localization accuracy and real-time efficiency in this context, supporting the design adopted in YOLOv8-CA.

**Figure 10:**
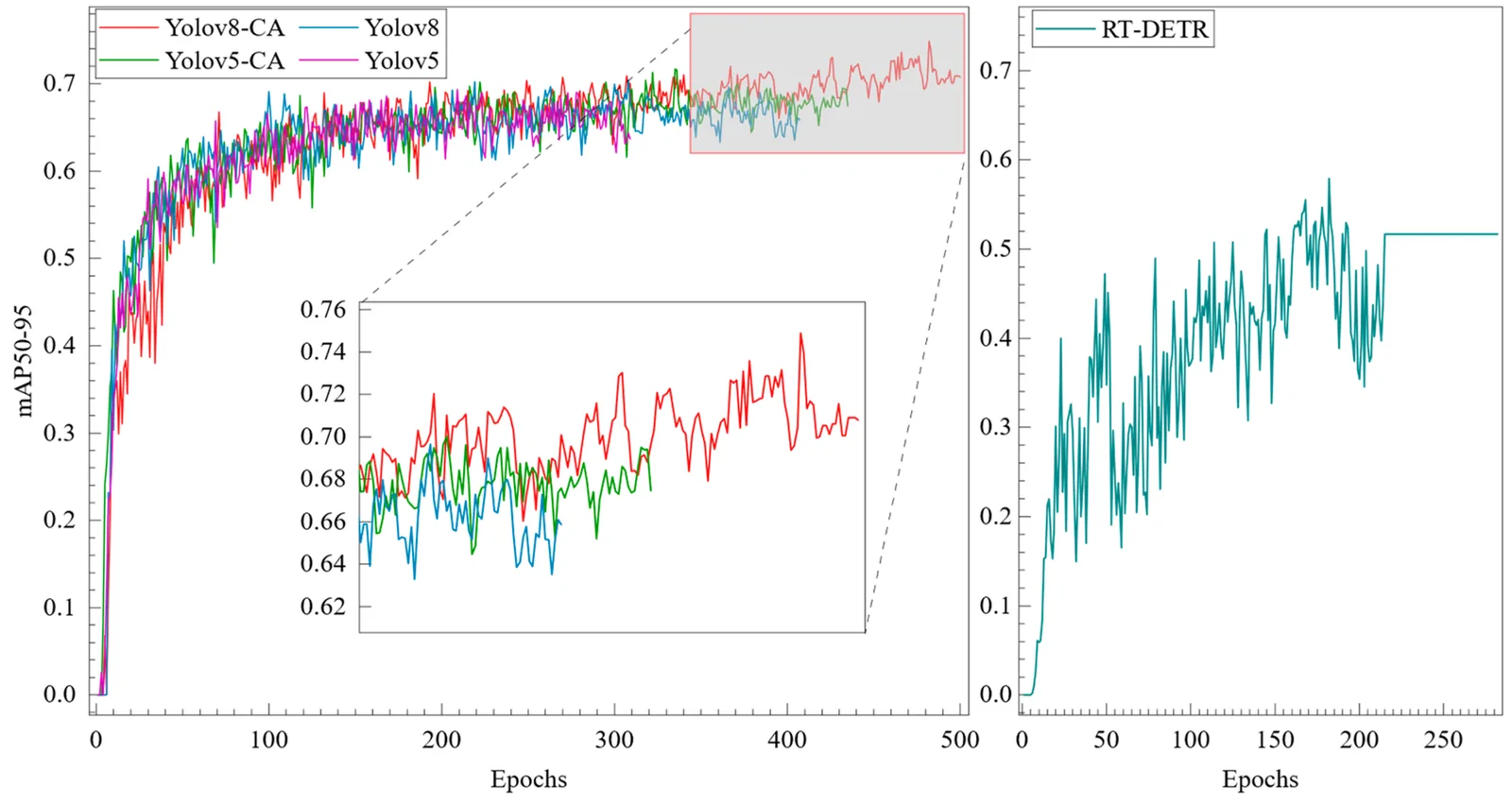
mAP50-95 comparison of visual detection models.

The training was conducted on a desktop equipped with an 8-core CPU and an NVIDIA RTX 4060 GPU. The models were pre-trained on the COCO dataset [50] to accelerate convergence and trained for 500 epochs, applying early stopping with a patience of 100 epochs. Figure 10 presents the mAP50-95 curves for each vision model, which had undergone independent hyperparameter fine-tuning processes to achieve optimal performance.

As shown in Figure 10, the curves for each model generally followed similar trends, but significant differences were observed in terms of stability and final performance. The YOLOv8-CA and YOLOv5-CA achieved higher peak and convergence mAP50-95 values compared to the original YOLOv8 and YOLOv5, while also exhibiting slower convergence. The training process of YOLOv8-CA reached the 500-epoch limit, indicating stable learning behavior and continuous improvement. The original YOLOv8 outperformed YOLOv5, while the integration of the CA module provided notable enhancements. In contrast, while RT-DETR has demonstrated competitive performance on general-purpose datasets such as COCO, its performance on the valve detection task reveals specific limitations related to the visual characteristics of this scenario, representing a task-specific gap rather than a general deficiency of the architecture. From the results, it may be inferred that the transformer-based global self-attention mechanism in RT-DETR captures long-range dependencies, which may result in a relatively dispersed attention distribution and weaken the emphasis on fine-grained local spatial features that are critical for detecting small-scale objects. Comparatively, YOLOv8-CA is designed to focus more on critical spatial regions and features relevant to small-scale objects, with the CA module enhancing spatial localization by explicitly encoding positional information along horizontal and vertical axes. Additionally, the training instability observed in RT-DETR suggests that the transformer architecture may require more hyperparameter tuning or larger datasets to converge effectively in this specific domain, whereas the proposed model demonstrated more robust convergence behavior. Overall, the results indicate that the proposed YOLOv8-CA outperformed the baselines, demonstrating improved performance in detecting small-scale components against varied backgrounds.

It should be noted that while a higher mAP50-95 indicates superior capability of the perception system, the comprehensive performance of the proposed robotic framework depends on the results of manipulation tasks, specifically on whether the detections lead to precise executions, which were evaluated in subsequent experiments.

After training and evaluation of the vision models, the best-performing weights were integrated into the visual perception system and adopted for subsequent experiments. The selected checkpoints with the corresponding metrics are summarized in Table 4.

It can be concluded more intuitively from Table 4 that the CA integration effectively improved visual detection. It is worth noting that the reported FPS values were measured for the vision models, rather than representing the full pipeline latency. In practical deployments, visual inference may benefit from cameras with higher frame rates and is coupled with motion planning and communication delays. The proposed vision model is expected to reserve sufficient computational and temporal budget for motion planning and other concurrent tasks while ensuring reliable detection capability, thereby attaining a balance between performance and inference speed. Overall, the YOLOv8-CA achieved the highest performance and maintained satisfactory inference speed, validating the methodology of the visual perception system and demonstrating its potential for the specified operational task.

### 4.3. Task Execution

To evaluate the overall performance of the framework, including the capability of comprehensive detection and manipulation with precision, and the adaptability to different software and hardware configurations, a series of manipulation experiments was conducted, with a focus on the success rate of task execution.

As detailed in Section 4.1, the experiments were conducted under different relative positions between the robotic arm and the target valve component, and the framework was evaluated across different hardware and vision model configurations. Specifically, each framework configuration was tested in 100 trials for setting 1 with fixed relative positions, and 300 trials for setting 2 with varied relative positions. A trial was considered successful only if the robotic arm could smoothly detect, reach, and adjust the valve without errors or collisions. The experimental results are summarized in Table 5.

In this experiment, the planning failures include both motion planning errors and low-confidence visual detections, where a detection is considered unreliable if the confidence score falls below the predefined threshold. On the other hand, failures that occur during the manipulation stage are recorded as execution deviations. To elaborate on this, each such deviation can be attributed either to one of the perception-related factors discussed above, including detector localization error, depth error, and residual hand–eye calibration bias, or to one of the manipulation factors, including collision, grasping failure, and rotational deviation in which the rotational displacement does not reach the target angle. It was observed that most of the execution deviations during evaluation result from the perception factors, while few arise from manipulation deviations. In the experiments with varied relative positions, the YOLOv8-CA configuration produced 16 (IRB 120) and 18 (UR3e) execution deviation failures, corresponding to roughly 5–6% of trials, whereas other configurations exhibited substantially more. This indicates that the CA-enhanced perception tightens the localization error distribution within the tolerance window, directly raising the success rate of task execution, and also confirms that perception accuracy, rather than motion execution, is the dominant source of execution deviations.

Regarding the propagation of perception-side uncertainties into the task execution results, the relevant factors primarily include depth acquisition from the RGB-D camera, hand–eye calibration accuracy, and localization error of the detected component. As the target position $P_{e}$ of the end-effector is obtained through the chain illustrated in Equation (4), a depth error ∆z is translated into a Cartesian error along the camera z-axis and is then propagated through the coordinate transformation pipeline. Additionally, the hand–eye transformation is derived from offline calibration. The camera is mounted on the fifth link of the robotic arm via a fixed joint, which minimizes the risk of collision and relative displacement, and the residual calibration error acts as a constant bias added to every localized point and shifts the grasping pose. In addition, the detector localizes the valve center with a pixel-level error, which becomes a spatial localization error in the operational frame after inverse projection. The framework tolerates these errors within bounded grasp windows, as noted in Section 4.1.

Generally, it can be observed that under the first setting, the proposed framework achieved consistent success with most configurations, indicating effective environmental noise resistance and high output consistency. In contrast, under the second setting with varied relative positions, the success rates decreased across all configurations, reflecting increased complexity of the task, where minor localization or manipulation errors could lead to failures. However, the results suggest that the CA integration significantly narrowed this gap. Specifically, both framework configurations with the CA module integrated maintained success rates around 90%, demonstrating satisfactory robustness, while other configurations experienced failures in over 20% of the trials. Additionally, our findings indicate that the configurations with RT-DETR were less effective, with higher occurrences of low-confidence detections and grasping deviations. Robotic arms with comparable specifications exhibited similar performance, demonstrating scalability. The results also highlight the satisfactory adaptability of the framework to variations in experimental scenarios for small-scale component detection and manipulation, reducing reliance on human intervention.

**Figure 11:**
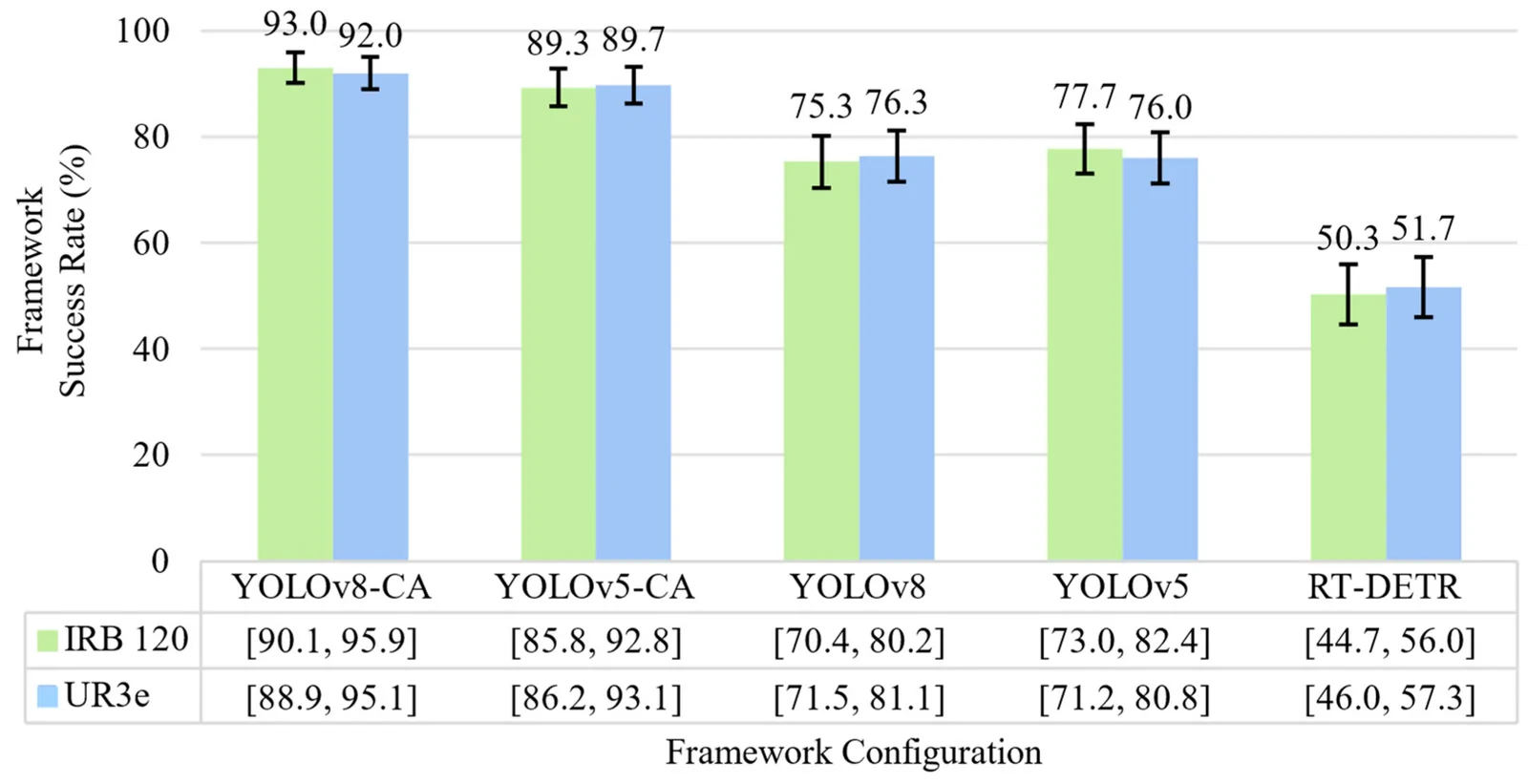
Success rates of task execution under random relative positions with 95% confidence intervals.

For the common practical scenario with random relative positions, a statistical analysis has been conducted following established experimental design principles [51]. Figure 11 presents the 95% confidence intervals for the framework’s success rate under each configuration. The results indicate that the framework can effectively integrate and leverage the strengths of different visual modules and hardware configurations, demonstrating its adaptability and extensibility under various operational conditions.

In summary, the experiments have confirmed the reliability of the proposed robotic framework for GIS gas pressure adjustment tasks. The results demonstrate success rates of over 90% under the proposed framework configuration in dynamic operational settings and exhibit adaptability to different software and hardware configurations, indicating potential for maintenance and operational applications, where precision, scalability, and collaborative autonomy are key considerations.

Despite these results, several limitations should be acknowledged. First, while the proposed framework is designed to be modular and expandable, the validated hardware options remain relatively constrained and could be further expanded. Second, although data augmentation, COCO pre-training, and random sensor noise are employed, some physical lighting variations such as glare are difficult to fully reproduce, and the limited dataset size and the single-source domain restrict the complexity and diversity of the training data. These limitations highlight the potential areas for future endeavors.

## 5. Conclusions

This paper presents a ROS-based robotic fine manipulation framework for high-precision collaborative autonomous operations in power infrastructure, evaluated on the task of gas pressure adjustment. With the integration of the CA module, the visual perception system demonstrates enhanced small-scale component detection. Furthermore, the motion control system supports accurate and smooth execution of manipulation commands, while the communication system enables seamless data interaction. The collaborative autonomy paradigm ensures safety and reliability in hazardous environments, enabling transitions between autonomous execution and remote human intervention as needed. Overall, the proposed framework enables collaborative autonomous detection and manipulation by robotic arm deployment in the GIS environment, demonstrating its potential for engineering operational applications.

Extensive experiments are conducted to evaluate the proposed robotic framework. The results confirm that the integration of the CA mechanism enhanced the performance of small-scale component detection, reducing execution deviation failures to roughly 5–6% of trials and yielding an approximately 15% improvement in task execution performance. In terms of the overall task success rate, the evaluations in static environments demonstrated satisfactory noise resistance and output consistency, while the trials in dynamic scenarios achieved a success rate of up to 93% with the proposed visual detector and IRB 120 configuration, validating the robustness and reliability of the framework, which confines human intervention to infrequent scenarios, thereby enhancing safety and minimizing operator workload.

This study proposes an integrated robotic framework beyond isolated algorithms and extends applied robotic capabilities from passive inspection to active manipulation of gas valves in hazardous power infrastructure. This work has the potential to advance infrastructure management and maintenance by providing a robotic alternative to manual operations. The modular design of the framework ensures flexibility and extensibility, enabling future integration with various robotic platforms and software techniques, laying the foundation for broader adoption of robotics in infrastructure.

Future research will focus on validating the framework’s compatibility with multiple robotic platforms, including FANUC and KUKA, and expanding the experimental scope to other operational tasks. Further domain randomization and synthetic-to-real fine-tuning will be implemented for enhanced generalization, and digital twin-based online calibration will also be applied to compensate for visual discrepancies between simulation and reality. Additionally, force and torque feedback will be explored for compliant control to handle non-ideal working conditions such as unexpected mechanical resistance. Lastly, deployment of the proposed framework in practical GIS scenarios and other infrastructure applications is planned for further validation and improvement.

## Figures and Tables

**Table 1:** Summary of the representative related studies with relative advantages and limitations.

Category	References	Relative Advantages	Relative Limitations
UAV-based platforms	[4,7,8,9,12,13,14]	Rapid coverage of wide areas and high accessibility to hard-to-reach areas	Limited payload, weather-dependent, and signal limitations in GPS-denied or confined spaces
UGV-based platforms	[3,10,11,15,16,17,18]	High payload capacity and stability, as well as suitability for confined spaces	Terrain restrictions, relatively slow speed, and limited coverage
Two-stage detectors	[23,24,25,26]	Strong performance in complex scenes and high localization accuracy	Slower inference speed, high computational cost, and challenges in real-time system deployment
One-stage detectors	[27,28,29,30,31]	Fast inference speed, suitability for real-time applications, and easier deployment on edge devices	Potential performance degradation in crowded scenes and sensitivity to class imbalance
RT-DETR (Vision Transformer-based)	[32,33,34,35]	Strong global context modeling and competitive performance on general datasets	Architectural complexity for edge deployment, higher sensitivity to hyperparameters and data scale, and potentially higher training cost
Attention mechanisms (SE, CBAM, ECA, SK, CA)	[39,40,41,42,43]	Enhance feature representations by dynamically recalibrating channel and/or spatial dimensions	SE and ECA lack spatial positional encoding, CBAM could have limited long-range interaction, SK shares fusion weights across all spatial positions, and CA may inadequately model non-axis-aligned 2D spatial correlations

**Table 2:** Predefined action commands for the robotic arm.

Action Name	Command Parameters	Description
/MoveJoints	joint 1, joint 2, …, joint 6 (rad), speed (rad/s)	Moves the robotic arm to a specific joint position
/MoveGP	delta (m), speed (m/s)	Moves the fingers of the gripper to a specific pose
/MoveLin	deltaX, deltaY, deltaZ (m), speed (m/s)	Moves the end-effector linearly along the specified Cartesian path
/MoveRot	yaw, pitch, roll (rad), speed (rad/s)	Rotates the end-effector with the specified Euler angles
/MovePos	x, y, z (m), speed (m/s)	Moves the end-effector to a specific position
/MoveEE	x, y, z (m), yaw, pitch, roll (rad), speed (m/s)	Moves the end-effector to a specific position and orientation

**Table 3:** Ablation studies on the insertion position of the CA module.

CA Insertion Position	mAP50-95	F1	FPS
Final backbone stage (the proposed YOLOv8-CA)	0.741	0.914	55.556
Backbone P4 (after C2f) and P5 (after SPPF)	0.719	0.908	53.251
Backbone P5 (after SPPF) and Neck P4 in both upsampling and downsampling paths (after C2f)	0.711	0.891	47.953
YOLOv8 baseline w/o CA	0.704	0.895	65.085
After each C2f stage in the backbone	0.703	0.881	45.105
Backbone P4 (after C2f)	0.699	0.916	60.669
Backbone P5 (after SPPF) and Neck P3 and P4 in the upsampling path (after C2f)	0.694	0.897	49.020
Neck P3 (after Concat)	0.676	0.899	59.336

**Table 4:** Metrics of the selected vision model checkpoints.

Model	mAP50-95	F1	FPS
YOLOv8-CA	0.741	0.914	55.556
YOLOv5-CA	0.717	0.896	49.866
YOLOv8	0.704	0.895	65.085
YOLOv5	0.694	0.879	55.230
RT-DETR	0.569	0.780	46.784

**Table 5:** Task execution results of the proposed framework.

Framework Configuration		Setting 1			Setting 2		
Robotic Arm	Vision Model Integration	Planning Failure	Execution Deviation	Success Rate (%)	Planning Failure	Execution Deviation	Success Rate (%)
IRB 120	YOLOv8-CA	0	0	100.0	5	16	93.0
	YOLOv5-CA				7	25	89.3
	YOLOv5				7	60	77.7
	YOLOv8				6	68	75.3
	RT-DETR	8	3	89.0	28	121	50.3
UR3e	YOLOv8-CA	0	0	100.0	6	18	92.0
	YOLOv5-CA				5	26	89.7
	YOLOv8				6	65	76.3
	YOLOv5				9	63	76.0
	RT-DETR	9	3	88.0	25	120	51.7

## Data Availability

The data presented in this study will be made available on request from the corresponding author.
